# Acoustic Comfort in Virtual Inner Yards with Various Building Facades

**DOI:** 10.3390/ijerph16020249

**Published:** 2019-01-16

**Authors:** Armin Taghipour, Tessa Sievers, Kurt Eggenschwiler

**Affiliations:** Empa, Swiss Federal Laboratories for Materials Science and Technology, Laboratory for Acoustics/Noise Control, Überlandstrasse 129, 8600 Dübendorf, Switzerland; tessa-sievers@gmx.de (T.S.); kurt.eggenschwiler@empa.ch (K.E.)

**Keywords:** acoustic comfort, inner yard acoustics, soundscape pleasantness, sound perception, virtual room acoustics, virtual audio, quality of experience

## Abstract

Housing complex residents in urban areas are not only confronted with typical noise sources, but also everyday life sounds, e.g., in the yards. Therefore, they might benefit from the increasing interest in soundscape design and acoustic comfort improvement. Three laboratory experiments (with repeated-measures complete block designs) are reported here, in which effects of several variables on short-term acoustic comfort were investigated. A virtual reference inner yard in the ODEON software environment was systematically modified by absorbers on building facades, whereby single-channel recordings were spatialized for a 2D playback in laboratory. Facade absorption was found, generally, to increase acoustic comfort. Too much absorption, however, was not found to be helpful. In the absence of any absorbers on the facade, absorbing balcony ceilings tended to improve acoustic comfort, however, non-significantly. Pleasant and unpleasant sounds were associated with comfort and discomfort, accordingly. This should encourage architects and acousticians to create comfortable inner yard sound environments, where pleasant and unpleasant sound occurrence probabilities are designed to be high and low, respectively. Furthermore, significant differences were observed between acoustic comfort at distinct observer positions, which could be exploited when designing inner yards.

## 1. Introduction

Urban living in areas with a high population density is affected by classic noise sources, such as road traffic, railways, and aircraft noise, as well as by surrounding everyday life sounds. Conventional environmental and construction acoustics deal with noise assessment and noise control with the goal to reduce the noise immission at the living space of the residents [1,2,3]. Beyond the topic of noise immission, however, there has been an increasing interest in soundscape design with the aim to improve the soundscape and acoustic comfort [4,5,6,7,8]. Despite some overlap between both topics, the main goal of soundscape design and acoustic comfort improvement is not only to reduce relatively unpleasant sounds (such as noise), but also to design and enable a relatively pleasant sound environment. Residents of housing complexes might benefit from the latter, e.g., by controlled absorptions and reflections of the facade. Whereas sound absorbing materials have been frequently used to improve acoustic comfort in closed rooms [9,10,11], little is known about the influences of sound absorbers on the (outside) facade to improve acoustic comfort for residents of housing complexes with shared inner yards.

One of the challenges in soundscape and acoustic design for housing complexes is that it involves collaboration of people from various backgrounds, such as acousticians, engineers, planners, architects, urbanists and sound quality experts, who—based on their different backgrounds—not only have different understandings of acoustic phenomena related to such a housing project, but also, sometimes, different goals [12,13,14]. Even the definition of the term “acoustic comfort” is vague and colorful in the literature. While, in many studies, an “improvement in acoustic comfort” meant a general improvement of acoustics and was measured by different sets of objective acoustical or room acoustical parameters (such as lower sound pressure level, SPL) [10,11], other studies used a subjective evaluation of acoustic comfort [9,15,16]. Furthermore, a series of studies measured an improvement in acoustic comfort directly with a decrease in noise annoyance (associated with corresponding decrease in SPL) [17]. While acoustic comfort has been found to be related to the SPL—i.e., $L_{\mathrm{eq}}$ (equivalent continuous sound level) or $L_{\mathrm{dn}}$ (day-night sound level)—[15], reducing SPL alone might not be a sufficient measure for improving acoustic comfort in urban areas [15,18,19]. Acoustic comfort seems to be rather determined by more factors than just the sound level [15,16]. For a review of the variety of definitions, descriptions, and indicators of acoustic comfort, see [20,21,22]. The study reported in this paper uses a subjective evaluation of acoustic comfort and discomfort.

Similar to other urban areas around the world, also in Switzerland, soundscape evaluation and improvement of acoustic comfort have gained interest [14,23,24,25] and the problem of finding a unique language, understanding, and goals between people from different backgrounds is evident. One of the areas under investigation is sounds in inner yards, whereby examples of inner yards of housing complexes have been investigated in order to find capacities for improvement of the sound environment and acoustic comfort [14].

In housing complexes, the presence of buildings induces complicating acoustic effects such as multiple reflections, diffraction, and diffusion, which depend, among others, on the material properties of building facades [26]. By using acoustic absorbers, for example, SPL in outside areas of housing complexes might be reduced [27], which can increase acoustic comfort. Compared to the semi-free field situation, Yang et al. [26] reported up to 8 dB increase in SPL due to multiple reflections outside of an apartment complex. This indicates potential acoustic performance improvements.

Yu and Kang [28] investigated sustainability of different facade materials on several building types with the aim to reduce the (negative) environmental impact while reserving similar acoustic performance. It was found that suitable selection of acoustic material can increase sustainability. Given that the aim of the study was to investigate sustainability at comparable levels of acoustic performance—and not to improve acoustic performance–, only negligible acoustical differences were reported between usage of different materials.

Generally, building facade absorption was found to affect acoustics of the outer space, for example, at public squares [27] or alongside streets [29,30]. Combined with a proper balcony design, building facades seem to be effective in mitigating noise immission [29]. Tang [31] reviewed complex behavior of facade balconies with different designs, whereby absorbers were found to be significant or insignificant based on their position and angle [31,32]. With a noise source on the ground or from the roadway, generally, balconies on a building facade provide significant acoustical protection to the areas beyond them [31,33]. This protective effect is, however, partially canceled by reflecting balcony ceilings [34,35]. In addition, multiple rectangular balconies were found to be problematic reflectors [32].

The above literature review shows a potential to improve acoustic performance (also) for housing complexes with inner yards. There seems to be a need to link the design (and the physical or acoustical parameters) directly to the acoustic comfort, rather than to the SPL. Possible contributions of absorbing materials on facades and balcony ceilings in enhancing acoustic comfort should be investigated.

This paper deals with short-term (momentary) acoustic comfort in the presence of sound from an inner yard in a housing complex. The study was carried out by means of three experiments, whereby only acoustic stimuli and no visual representation was used. Laboratory experiments with audio [36,37,38,39,40,41] or audio-visual [17,42,43,44] stimuli have been increasingly used to investigate questions related to noise annoyance, soundscape, and/or acoustic comfort, partially because models of reality are extremely helpful in the planning and design stage [44]. Furthermore, for comparison studies, laboratory experiments offer a controlled setup, which is not easily practicable in the field. It should be mentioned that portions of this study were Presented at the DAGA 2018 (44. Jahrestagung für Akustik) [45], Munich, Germany, 19–22 March 2018.

Section 2 and Section 3 of this paper give an introduction to the experimental concept, design and setup. Three experiments and their results are presented in Section 4, Section 5 and Section 6. Section 7 and Section 8 offer a discussion of the results and conclude the paper.

## 2. Experimental Concept

In this study, short-term or acute comfort and discomfort reactions to sounds in virtual acoustic rooms were investigated under laboratory conditions. The term “short-term” refers to the time period during and after an acoustic stimulus’ playback and before the next stimulus is presented. The observed “short-term” comfort or discomfort ratings were, hence, related to acute comfort in response to each stimulus, rather than long-term comfort or well-being.

To investigate possible differences in short-term comfort in inner yards with distinct building facades, sound propagation was simulated in virtual rooms, whereby single-channel recordings were auralized for a multi-channel playback system (see Section 3). To that aim, several design variables, such as categorical variables sound source type and observer position as well as continuous variables such as absorption coefficient were systematically varied to study their individual and combined associations with short-term acoustic comfort ratings. Observer positions were either outside in the yard, outside on the balcony or at open windows. That is, inner room acoustics (such as in [28]) were not investigated here.

The authors would also like to note that throughout this paper the terminology “acoustic comfort” will be used. That is, the German terminology in the questionnaire *sich wohl fühlen* was translated to “to feel comfortable” or “to be comfortable.”

## 3. Experimental Design and Setup

This section reports a subset of the information related to the experimental setup and method, which was similar for all three experiments. Further method-related information specific to each experiment (such as stimuli or subjects) will be reported later in the respective sections.

### 3.1. Listening Test Facility

The experiments presented in this paper were conducted in the listening test facility of the Empa (Swiss Federal Laboratories for Materials Science and Technology), named AuraLab, which has a separate listening and control room allowing for audio-visual supervision to comply with ethical requirements.

**Figure 1:**
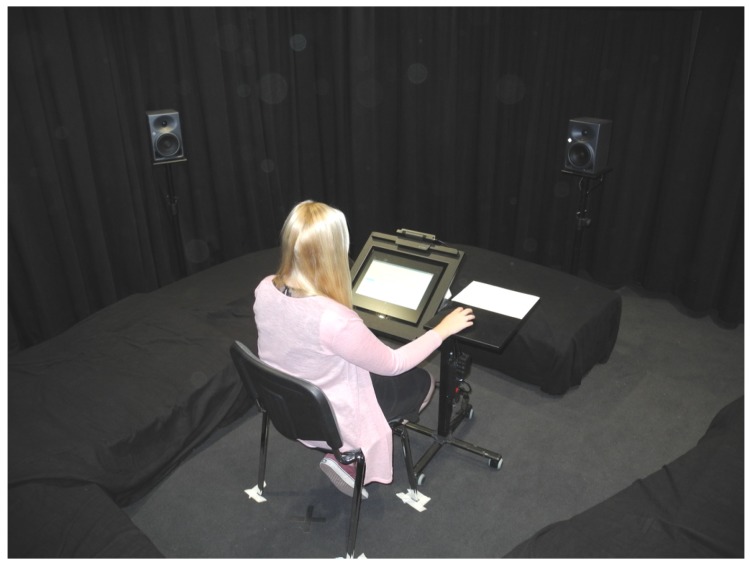
Experimental setup in AuraLab with five loudspeakers and additional porous floor absorbers.

AuraLab satisfies room acoustical requirements for high-quality audio reproduction in terms of its background noise and reverberation time. For the present experiments, the carpeted floor was covered with additional absorbers on the floor. The experimental setup in AuraLab is shown in Figure 1. Subjects were seated in the central listening spot. A 3D immersive sound system with 16 separate audio channels is installed in AuraLab. Fifteen loudspeakers “KH 120 A” (Georg Neumann GmbH, Berlin, Germany) are located in a hemispherical arrangement on three levels (0, 30, and 60° vertically) at a distance of 2 m from the central listening spot. Bass management by means of two subwoofers “KH 805” (Georg Neumann GmbH, Berlin, Germany) and a digital signal processor complete the playback system. Stimuli of the experiment presented here were played back by a 2D setup over the five loudspeakers at 0° elevation (subject’s ear level) and both subwoofers.

### 3.2. Signal Processing and Room Acoustic Simulation

**Figure 2:**
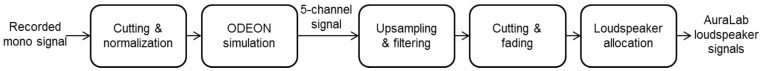
Block diagram of the signal processing steps from recording to playback.

A block diagram of signal processing—from recording to playback—is given in Figure 2. Single-channel recordings were carried out in a semi-anechoic chamber. A B&K 2006 microphone (Brüel & Kjaer, Nærum, Denmark) was positioned on the reflecting floor. Recorded individuals (or, in case of children, their parents) were informed about the purpose of the recordings and signed a consent form, which was prepared under supervision and with approval of Empa’s Ethics Committee Office.

Suitable 8-s extracts were made for the purpose of this study. The extracts were normalized to the A-weighted level (i.e., A-weighted equivalent continuous sound level, $L_{\mathrm{Aeq}}$) of the signal with the largest maximum absolute amplitude.

Room acoustic simulations were done with ODEON software version 14.03 (Odeon A/S, Kgs. Lyngby, Denmark), which uses the image-source method and a ray tracing algorithm. A 2D auralization (2D surround sound) was chosen for five loudspeakers such as the AuraLab setup. Although the ODEON input signals (of different sources) had the same $L_{\mathrm{Aeq}}$ (see above), the ODEON outputs exhibited diverging $L_{\mathrm{Aeq}}$, as they possessed unequal spectral and temporal characteristics, which reacted differently to the virtual rooms. The stimuli reported in this paper were simulated considering a single source, one source position, and several observer positions.

The multi-channel ODEON output signal was upsampled from 44.1 to 48 kHz, as this is a requirement of the playback system. It was then low pass ($f_{c}=10$ kHz) and high pass ($f_{c}=20$ Hz) filtered. After being gated with squared-cosine ramps, the multi-channel signal was allocated to the corresponding loudspeakers: front, front-left, front-right, back-left, and back-right.

All the processing steps (with exception of ODEON simulations) were carried out with MATLAB R2016b (MathWorks, Natick, MA, USA).

### 3.3. Reference Inner Yard

**Figure 3:**
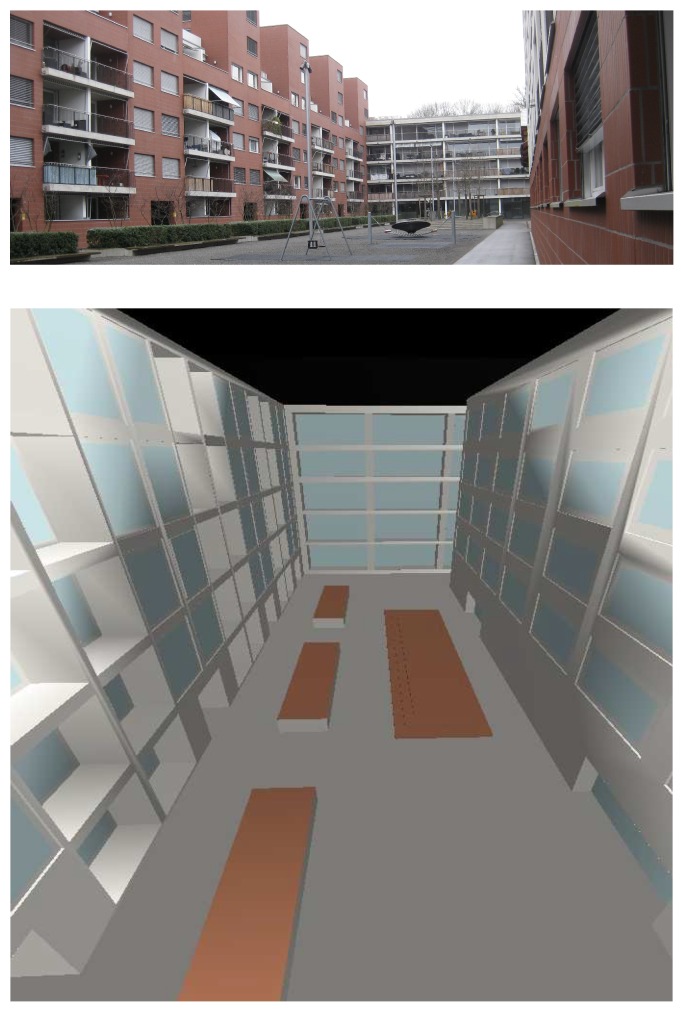
Reference inner yard in original (**above**) and as a simplified geometric model for the ODEON room acoustic simulation (**below**). Absorption coefficient of the materials is coded by their colors, whereby, black stands for the fully absorbing encasing box. Blue and gray represent the reflecting glass or brickwork facade, respectively. The floor is either reflecting smooth unpainted concrete (gray) or absorbing grass (brown).

The reference inner yard used in the course of this study was a simplified 3D model of an existing housing complex in Dübendorf, Switzerland (see Figure 3). The geometric model was built in the SketchUp software environment (Trimble Inc., Sunnyvale, CA, USA) and was imported into the ODEON software environment using the plug-in SU2Odeon. The walls were of brick with large glass windows. Since ODEON works with bounded/closed room models, the inner yard was modelled as a ceiling-less room (100 m × 20 m × 20 m), which was inserted in a larger box (10 m away from each side) with a perfect absorbing inner surface, representing free field.

### 3.4. Validation

In order to validate the system (i.e., the simulation-playback chain), a series of evaluation tests was carried out in AuraLab. Sound pressure levels ($L_{\mathrm{Aeq}}$, $L_{\mathrm{AFmax}}$), level reductions for several simulated distances (from a point source), and localization tests were carried out for single and/or multiple source(s) and several virtual source positions. This was done either by level measurement in AuraLab or, in the case of the source localization tests, by five expert listeners, who were acousticians.

### 3.5. Experimental Sessions

All three experiments were conducted as focused listening tests with repeated measures and a complete block design; i.e., each subject listened to all the stimuli of the experiment they participated in and rated the acoustic comfort they associated with each stimulus during or directly after its playback.

Subjects did the tests individually (i.e., one by one). After reading study information, they signed a consent form to participate, after which they answered the first part of the questionnaire about hearing and well-being. The subjects were then introduced to the listening test and the test software which guided them through the test. After the listening test, the subjects filled out the rest of the questionnaire (demographic data).

Whereas Experiment 1 was conducted as a single listening test, Experiments 2 and 3 were conducted as two listening tests in one experimental session (after each other). That is, there were two groups of subjects: those who participated in Experiment 1 and those who participated in Experiments 2 and 3. For the latter experimental session, the order of the Experiments 2 and 3 was (randomly) counterbalanced between the subjects.

### 3.6. Listening Test Software, Procedure, and the Comfort Scale

The listening tests were done by means of a software (and its graphical user interface) which guided the subjects through the test. To familiarize the subjects with the sounds (incl. virtual rooms) and the test software, the subjects listened to several orienting and training stimuli. The main listening test began thereafter. For each stimulus, subjects completed the following statement: “In this virtual inner yard and in the presence of this sound, I feel …”. Their short-term acoustic comfort was recorded during or after stimulus playback on a verbal bipolar 7-point scale: very uncomfortable (−3), uncomfortable (−2), to some extent uncomfortable (−1), neither comfortable nor uncomfortable (0), to some extent comfortable (+1), comfortable (+2), and very comfortable (+3). For the statistical analysis, the ratings were coded from −3 to 3, respectively.

To support the neutral category “neither uncomfortable nor comfortable” in its actual function as the scale center and to avoid its misuse as an avoiding or diverting answer [46], an additional “don’t know” push button was made available to the subjects. This option was, however, never used by the subjects.

The stimuli were played in a random order after one another, with a 1.2-s break between stimuli after complete playback. By means of a push button, an option was given to the subjects to listen to each stimulus (only) one more time, if they wished to. The subjects rarely made use of this option.

### 3.7. Pilot Experiments

Prior to the experiments presented here, a number of pilot tests were carried out, based on which the number of stimuli, the selection of stimuli, and minor changes in the design were carried out. To avoid an unnecessarily long text, the design and the results of the pilot tests will not be reported in this paper. Effort was made to ensure that subjects from the pilot test, as well as other people aware of the study, did not participate in the main experiments. Fewer than 10% of interested pilot subjects were allowed to participate in the main experiments.

### 3.8. Statistical Analysis

The statistical analysis was carried out with IBM SPSS Statistics, version 25 (IBM Corporation, Armonk, NY, USA). Tested effects were considered significant if the probability, *p*, of the observed results under the null hypothesis (H0) was ≤0.05.

The individual and combined associations of the independent variables (i.e., experimental design variables) on short-term acoustic comfort was investigated as follows:

- Repeated-measures multi-factorial analysis of variance (ANOVA), corrected by the Greenhouse–Geisser method.
- Post hoc pairwise comparisons by Fisher’s protected least significant difference (LSD) test, corrected by the Bonferroni method.
- Linear mixed-effects models for further combined analysis of variables of different types; i.e., categorical variables, covariates, and random intercept (comparison of the models by means of Akaike information criterion (AIC) [47] and Bayesian information criterion (BIC) [48]; i.e., choosing the model with the lowest AIC/BIC).

## 4. Experiment 1

Before testing different absorbing materials on the building facade, Experiment 1 dealt with the question, whether usage of (any) absorbers on the facade would change the inner yard soundscape such that the subjective acoustic comfort would be affected. There is evidence for such an effect in the case of outer spaces in urban areas (a small urban square) [27]. Furthermore, it was to assume that, at a given sound level, subjects would have a different perception of relatively pleasant and unpleasant sounds [15]. Therefore, since—in contrast to the unipolar (negative) 11-point ICBEN (International Commission on Biological Effects of Noise) noise annoyance scale [49]—the bipolar 7-point comfort/discomfort scale was to be associated with comfort (i.e., positive) or discomfort (i.e., negative), relatively pleasant as well as relatively unpleasant stimuli were presented to the subjects. Relative pleasantness and unpleasantness of sounds was evaluated after a statistical analysis of data of a pilot experiment (not reported here) and by environmental acousticians. The chosen sounds throughout this study were aimed to be representative for human-made (i.e., neighborhood) sounds occurring in typical inner yards.

### 4.1. Method

#### 4.1.1. Hypotheses

The following three experimental hypotheses were to be tested in Experiment 1 [45]:

- Facade changes affect short-term acoustic comfort.
- Short-term acoustic comfort depends on sound source.
- Short-term acoustic comfort depends on observer position.

#### 4.1.2. Experimental Design

Three design variables (i.e., independent variables) were considered in the design of Experiment 1: inner yard (four levels), source type (five levels), and observer position (three levels) [45].

Four inner yards were modelled in ODEON: the **reference** inner yard, an inner yard with one complete (long) side of the housing complex covered with a reflecting **glass facade**, an inner yard with **absorbing facade**, and an inner yard with **exaggerated absorption** of the housing complex whereby a number of glass windows and doors on the ground floor were covered by absorbing materials. Five different sources were used: a bouncing ball (**basketball 1**), a **doll’s pram** (played/pushed by a child), a German conversation (**conversation 1**), and two sounds of (happily) playing and laughing children (**children 1** and **children 2**). An omni-directional sound source was placed in the yard 1.2 m above the ground. Three observer points were chosen: two observer points in the yard (1.2 m above the ground, representing the position of someone sitting on a bench), **5** and **20** m away from the source, and one observer point on a balcony (**balcony 2**) about 25 m away from the source (second floor, 9 m above the ground). Note that, on average, $L_{\mathrm{Aeq},\mathrm{Balcony}2}<L_{\mathrm{Aeq},20m}<L_{\mathrm{Aeq},5m}$ and that the observer position at the balcony was considerably more affected by echos and flutter echos.

#### 4.1.3. Stimuli

Based on the combination of four inner yards, five sources, and three observer positions, a total number of 60 stimuli were prepared for this experiment: 4 × 5 × 3 = 60. It was mentioned that the input signals for the auralization in ODEON were 8 s long. Because of the reverberation time in the simulated inner yards, ODEON output audio files were of a length of up to 11 seconds. To have a unique length for all the stimuli used in the experiment, all ODEON outputs were cut down to 9 s. Fade-in and -out was realized with squared-cosine ramps of 200 and 600 ms, respectively. A-weighted equivalent continuous sound levels, $L_{\mathrm{Aeq}}$, were between 42 to 59 dB(A) (mean $L_{\mathrm{Aeq}}=52$ dB(A)).

#### 4.1.4. Subjects

Twenty-seven subjects (7 females and 20 males) participated in Experiment 1. All subjects declared to have normal hearing (self judgment) and to feel well. They were aged between 19 and 57 years (median 38 years). The majority of the participants worked at the Empa.

### 4.2. Results

**Figure 4:**
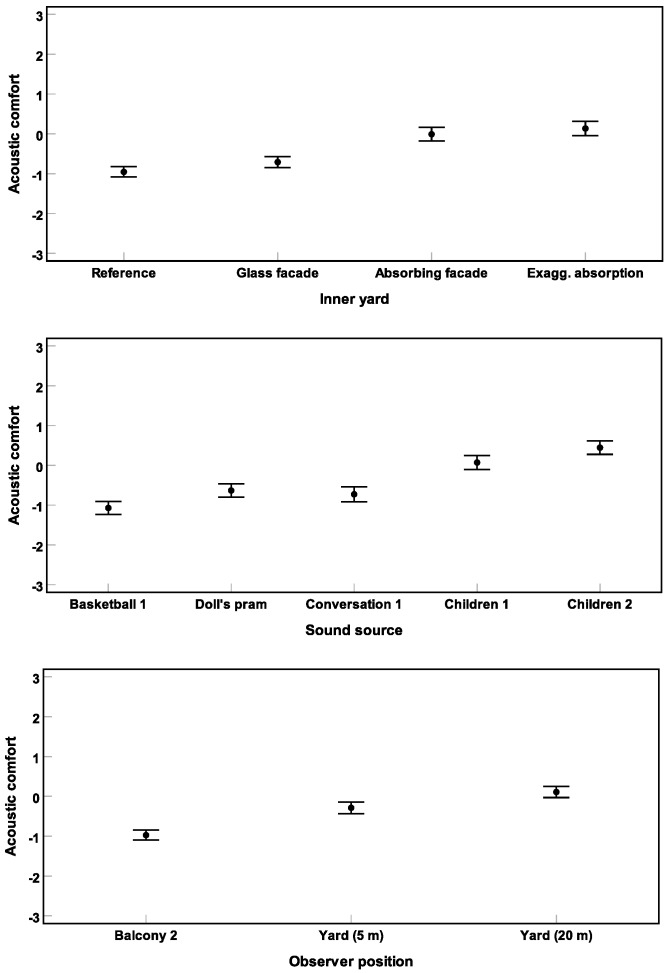
Results of Experiment 1: Mean acoustic comfort ratings across subjects and their 95% confidence intervals are shown for different inner yards (**above**), sound sources (**middle**), and observer positions (**below**).

Figure 4 shows the results of Experiment 1. A repeated-measures three-way ANOVA (corrected by the Greenhouse–Geisser method) revealed significant main effects of all three design variables inner yard (F(2.2, 57.2) = 49.6), sound source (F(1.2, 31.9) = 25.8), and observer position (F(3.1, 79.3) = 33.1) on short-term acoustic comfort (all p<0.001) [45].

Post hoc pairwise comparisons by an LSD test (corrected by the Bonferroni method) revealed no significant difference between the reference and glass facade yards (p>0.05). No significant difference was found between the two absorbing yards (p>0.05). However, in comparison to the non-absorbing yards, short-term acoustic comfort was rated significantly higher for the absorbing yards (all p<0.001). With respect to sound sources, pairwise comparisons revealed that short-term acoustic comfort was rated higher for the two **children** sounds than for the three other sound sources (all p<0.001). No further significant differences were found (all p>0.05). Furthermore, comfort ratings for the three observer positions found to be significantly different from each other (all p<0.01). The observer positions at 20 m distance in the yard and at the second floor balcony were found to be “more” and “less” comfortable than the position at 5 m distance in the yard, respectively (see Figure 4).

**Figure 5:**
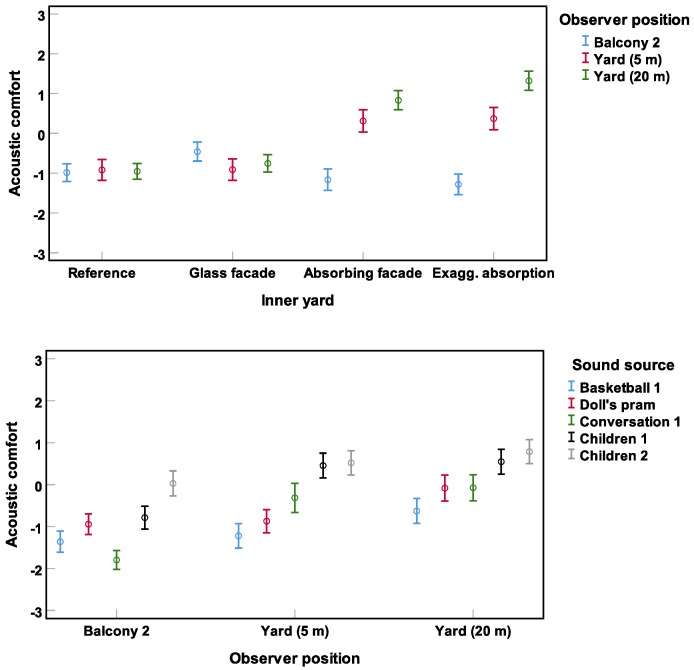
Significant interactions (Experiment 1) between inner yard and observer position (**above**) and between source and observer position (**below**). Mean acoustic comfort ratings across subjects and their 95% confidence intervals are depicted.

The repeated-measures ANOVA revealed two significant interactions between yard and position (F(3.8, 97.5) = 52.8) and between source and position (F(6.0, 155.4) = 11.5) (all p<0.001). These interactions are shown in Figure 5. Whereas absorption improved the acoustics—with respect to acoustic comfort—for the observer positions in the yard, the observer point on the balcony did not benefit from the absorbing materials. The balcony stimuli revealed much more flutter echo than for the other positions (The authors would like to report an error in the case of stimuli with one inner yard, namely the glass front, and one observer position, i.e., on the balcony. All five of these stimuli were, by mistake, set on the first balcony. That explains less flutter echo and higher acoustic comfort ratings in this case. Had this been correct, would the already significant differences—and the explanation given in the text—have been even stronger. Therefore, the authors can assure that this mistake did not affect the conclusions of this study.). The interaction between sound source and observer position indicates several points. First, whereas for the human sounds (conversation 1 and the two playing **children** stimuli), acoustic comfort ratings were similar at 5 m and 20 m away in the yard, they were significantly lower on the balcony, where more echo and more flutter echo was present. Second, for basketball 1 and doll’s pram—as examples of relatively unpleasant sounds—the 20 m position in the yard was significantly more comfortable than the two other observer positions. Note that, while $L_{\mathrm{Aeq},\mathrm{Balcony}2}<L_{\mathrm{Aeq},5m}$, at the balcony echo and flutter echo were more dominant. That is, only at the yard position 20 m away from the source both $L_{\mathrm{Aeq},20m}$ and echos or flatter echos were less dominant.

## 5. Experiment 2

The results from Experiment 1 showed that using absorbing materials on building facades increased short-term acoustic comfort. Experiment 2 aimed to focus on the effect of the absorption coefficient. 63% of the total facade of the reference building was non-glass material. This portion of the surface was varied from being a concrete reflecting facade to an absorbing facade. The main question under test was to what degree absorbing facade would improve acoustic comfort. Would there be saturation effects? Could too much absorption even lead to a decreased acoustic comfort?

### 5.1. Method

#### 5.1.1. Hypotheses

The following three experimental hypotheses were established for Experiment 2:

- Short-term acoustic comfort depends on the absorption coefficient.
- Short-term acoustic comfort depends on the sound source.
- Short-term acoustic comfort is different at different balconies (i.e., floors).

#### 5.1.2. Experimental Design

Three design variables (i.e., independent variables) were considered in the design of Experiment 2: the weighted absorption coefficient $\alpha_{w}$ [50] (five levels), source type (four levels), and observer location (two levels).

**Figure 6:**
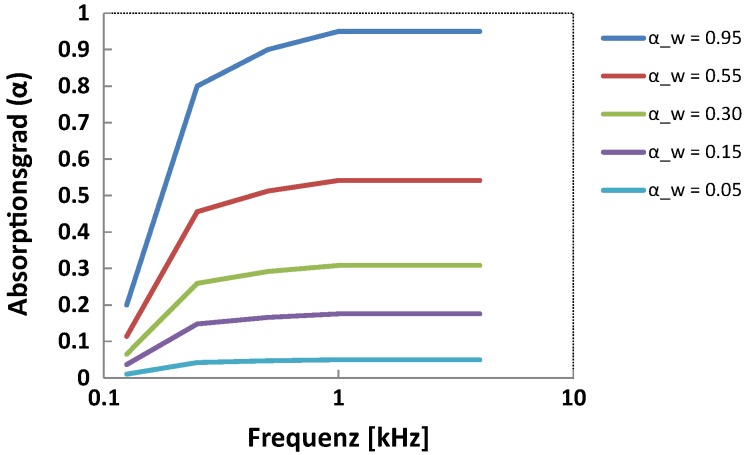
Absorption coefficient as a function of frequency (logarithmic horizontal axis).

The two modelled inner yards **reference** and **absorbing facade** in Section 4.1.2 exhibited $\alpha_{w}$ values of nearly 0 and 1. Having these two cases in mind as extreme (realistic) cases of reflection and absorption, $\alpha_{w}$ was varied for Experiment 2 from 0.05 to 0.95 with an approximately exponential progression (i.e., approximately equidistant on a logarithmic axis). In order to avoid major frequency-dependent differences in absorption properties of the materials—because that would have led to a tremendously more complicated interpretation of the results—a simple material model was chosen, for which the frequency dependency of α remained approximately constant, as $\alpha_{w}$ was increased. Figure 6 shows the absorption coefficient as a function of frequency as $\alpha_{w}$ increases. Doing so, the facade was covered with absorbing materials exhibiting $\alpha_{w}$ values of **0.05**, **0.15**, **0.30**, **0.55**, and **0.95**.

Four different sources were used: a bouncing ball (**basketball 1**), a crying **baby**, a Swiss German conversation (**conversation 2**), and a sound of playing children (**children 2**). As in Experiment 1, an omni-directional sound source was placed in the yard 1.2 m above the ground. Two observer points were chosen on the **balcony 0** (patio) and **balcony 2** (second floor balcony). Compared to the level at **balcony 0**, **balcony 2** exhibited lower $L_{\mathrm{Aeq}}$ (i.e., mean $L_{\mathrm{Aeq},\mathrm{balcony}2}<$ mean $L_{\mathrm{Aeq},\mathrm{balcony}0}$).

#### 5.1.3. Stimuli

Based on the combination of five absorption degrees ($\alpha_{w}$), four sources, and two observer positions, a total number of 40 stimuli were prepared for Experiment 2: 4 × 5 × 2 = 40. ODEON outputs were cut down to 10 s. Fade-in and -out was realized with squared-cosine ramps of 400 and 1000 ms, respectively. A-weighted equivalent continuous sound levels, $L_{\mathrm{Aeq}}$, were between 49 to 64 dB(A) (mean $L_{\mathrm{Aeq}}=60$ dB(A)).

#### 5.1.4. Subjects

Forty-two subjects (13 females and 29 males) participated in Experiment 2. All subjects declared having normal hearing (self judgment) and to feel well. They were aged between 18 and 64 years (median 41 years). The majority of the participants worked at the Empa.

### 5.2. Results

**Figure 7:**
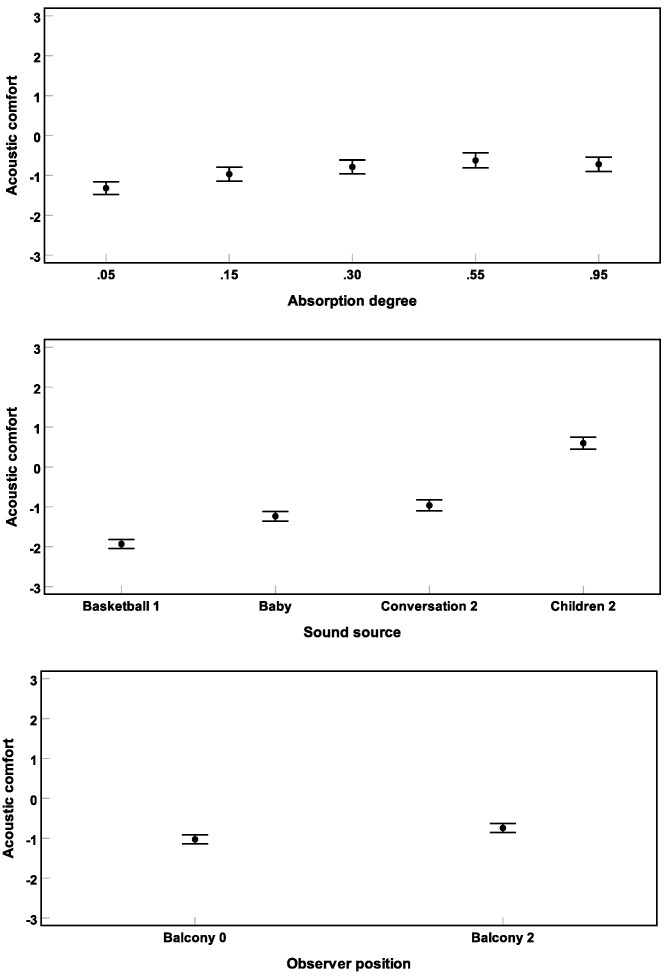
Results of Experiment 2: Mean acoustic comfort ratings across subjects and their 95% confidence intervals are shown for different absorption degrees $\alpha_{w}$ (**above**), sound sources (**middle**), and observer positions (**below**).

Figure 7 shows the results of Experiment 2. A repeated-measures three-way ANOVA (corrected by the Greenhouse–Geisser method) revealed significant main effects of all three design variables $\alpha_{w}$ (F(3.0, 121.8) = 21.0), sound source (F(2.7, 112.1) = 71.6), and observer position (F(1.0, 41.0) = 29.3) on short-term acoustic comfort (all p<0.001).

**Figure 8:**
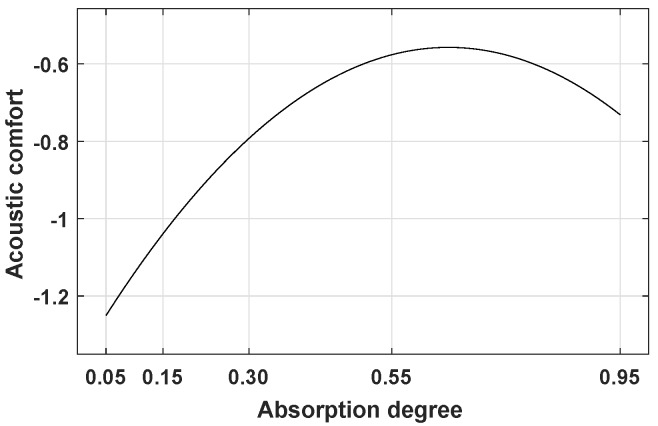
Effect of the absorption coefficient ($\alpha_{w}$) on short-term acoustic comfort, estimated by a linear mixed-effects model.

A linear mixed-effect model was established considering the covariates absorption coefficient ($\alpha_{w}$) and playback number, categorical variables sound source and observer position, as well as random intercept. Since the effect of $\alpha_{w}$ seemed to have a parabolic shape here (and in a pilot experiment), also ${\alpha_{w}}^{2}$ was considered in the model. The estimate of the model for the effect of $\alpha_{w}$ is depicted in Figure 8.

Post hoc pairwise comparisons by LSD test (corrected by the Bonferroni method) revealed that **basketball 1** and **children 2** were rated as least and most comfortable sounds, respectively (all p<0.01). No significant difference was found between the ratings for crying **baby** and for the **conversation 2** (p>0.05). Furthermore, acoustic comfort was rated higher for **balcony 2** than for **balcony 0** (p<0.01).

**Figure 9:**
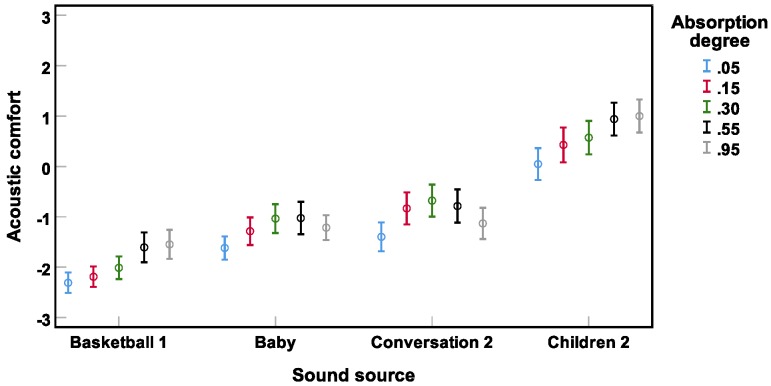
Significant interaction (Experiment 2) between $\alpha_{w}$ and sound source. Mean acoustic comfort ratings across subjects and their 95% confidence intervals are depicted.

The repeated-measures ANOVA revealed a significant interaction between $\alpha_{w}$ and sound source (F(7.3, 301.3) = 4.7) (p<0.001), which can be observed in Figure 9. Whereas for **basketball 1** and **children 2** acoustic comfort was increased with increasing $\alpha_{w}$ up to a saturation effect for $\alpha_{w}>0.55$, for crying **baby** and **conversation 2**, a strong parabolic relationship was found between $\alpha_{w}$ and acoustic comfort.

## 6. Experiment 3

In Experiment 3, possible effects of the absorption of balconies’ ceilings on acoustic comfort was investigated. Thus, not only facade absorption was varied, but also every balcony ceiling.

### 6.1. Method

#### 6.1.1. Hypotheses

In Experiment 3, the following three experimental hypotheses were investigated:

- Short-term acoustic comfort depends on the absorption coefficient ($\alpha_{w}$).
- Short-term acoustic comfort depends on the sound source.
- Using absorbing materials on the balcony ceiling affects short-term acoustic comfort.

#### 6.1.2. Experimental Design

Three design variables were considered in the design of Experiment 3: facade $\alpha_{w}$ (three levels), source type (three levels), and balcony ceiling $\alpha_{w}$ (three levels). $\alpha_{w}$ was varied for Experiment 3 between **0.05**, **0.30**, and **0.95** for the absorption of the facade, as well as the balcony ceiling. Three sound sources were used: a bouncing ball (**basketball 2**), **conversation 1**, and **children 1**. The observer was placed at the second floor balcony.

#### 6.1.3. Stimuli

Based on the combination of three facade absorption degrees, three sources, and three balcony ceiling absorption degrees, a total number of 27 stimuli were prepared for Experiment 3: 3 × 3 × 3 = 27. ODEON outputs were cut down to 10 s with fade-in and -out ramps of 400 and 800 ms, respectively. A-weighted equivalent continuous sound levels, $L_{\mathrm{Aeq}}$, were between 49 to 59 dB(A) (mean $L_{\mathrm{Aeq}}=56$ dB(A)).

#### 6.1.4. Subjects

Experiments 2 and 3 were conducted in one experimental session (see Section 3.5). The same subjects as in Section 5.1.4 participated in Experiment 3.

### 6.2. Results

**Figure 10:**
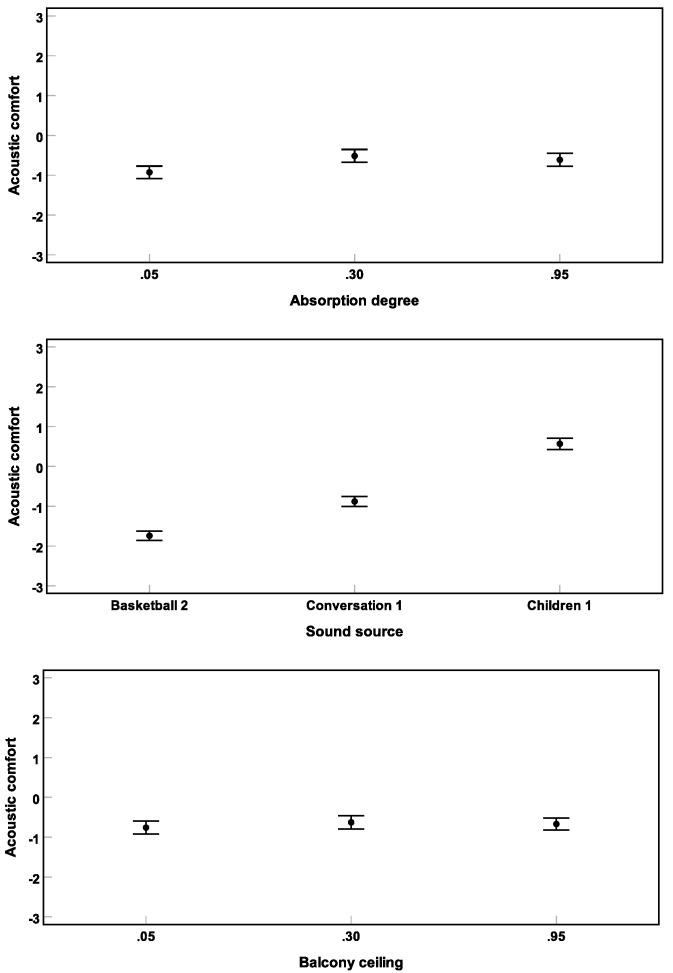
Results of Experiment 3: Mean acoustic comfort ratings across subjects and their 95% confidence intervals are shown for different absorption degrees $\alpha_{w}$ (**above**), sound sources (**middle**), and absorption degrees of the balcony ceiling $\alpha_{w}$ (**below**).

Figure 10 shows the results of Experiment 3. A repeated-measures three-way ANOVA (corrected by the Greenhouse–Geisser method) revealed significant main effects of facade $\alpha_{w}$ (F(1.4, 56.6) = 9.5) and sound source (F(2.0, 80.0) = 105.7) on short-term acoustic comfort (all p<0.01). $\alpha_{w}$ at the balcony ceiling was not found to be significantly affecting acoustic comfort (F(2.0, 80.3) = 2.8) (p=0.07).

A linear mixed-effect model was established considering the covariate facade $\alpha_{w}$ (and ${\alpha_{w}}^{2}$), and the categorical variable sound source, as well as random intercept. Contribution of balcony ceiling $\alpha_{w}$ (and/or ${\alpha_{w}}^{2}$) in the model were not significant. Even though only three absorption degrees were tested in Experiment 3, the model estimate was very similar to the estimate in Experiment 2. Regarding sound source, post hoc pairwise comparisons revealed that **basketball 2** and **children 1** were rated as “least” and “most” comfortable sounds, respectively (all p<0.001).

**Figure 11:**
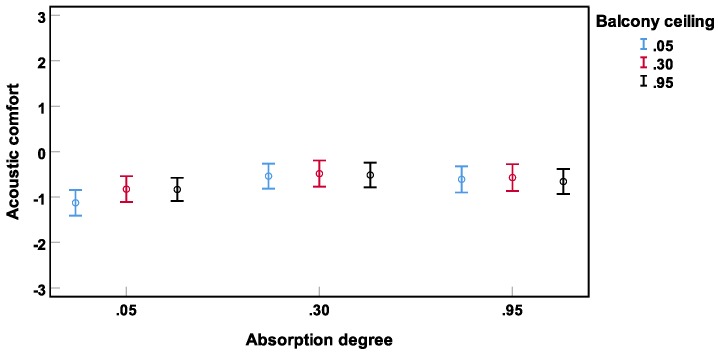
Non-significant interaction (Experiment 3) between $\alpha_{w}$ on the facade and $\alpha_{w}$ on the balcony ceilings. Mean acoustic comfort ratings across subjects and their 95% confidence intervals are depicted.

No interactions between the independent variables were found to be significant in predicting acoustic comfort. However, the authors would like to note that similar to the tendency for the main effect of balcony ceiling $\alpha_{w}$ (p=0.07; see above), there was a tendency for an interaction between $\alpha_{w}$ at the facade and at the ceiling (p=0.09). Thereby, for facade $\alpha_{w}$ of **0.05** (reflecting facade), usage of absorber at the balcony ceiling tended to increase acoustic comfort (see Figure 11). That is, only if there were no absorption on the walls, the absorbing material on the ceiling tended to be effective, however non-significant.

## 7. Discussion

The results of Experiment 1 revealed that the inner yards with absorbing building facades were acoustically more comfortable than the inner yards with reflecting building facades (see Section 4.2). Using a complete **glass facade** instead of the original brickwork and glass mix (in the **reference**) did not lead to significant changes in the acoustic comfort ratings. Similarly, an **exaggerated absorption** was not found to be significantly more effective than a simple **absorbing facade**. The results of Experiment 2 showed a similar pattern, whereby acoustic comfort increased with increasing $\alpha_{w}$, however only up to $\alpha_{w}=0.55$. Stronger absorption led to even lower acoustic comfort ratings (see Section 5.2).

Including ${\alpha_{w}}^{2}$ in the regression model and the resulting parabolic predicting relationships between $\alpha_{w}$ and short-term acoustic comfort seem to have provided a reasonable fit to the data (see Section 5.2). For the data presented here, only one point ($\alpha_{w}=0.95$) fell on the right side of the parabolic curve. One should be careful about whether one point justifies such a model suggestion. However, not only could a similar curve be fitted to the data from Experiment 3 (see Figure 10 (above)), but also the results of a pilot test (not presented here), which included $\alpha_{w}=1.00$, confirmed the parabolic shape. Thus, it is to assume that using absorbing materials on the facade would improve acoustic comfort, but only up to a point (for Experiment 1: the inner yard with **absorbing facade**; for Experiment 2: $\alpha_{w}=0.55$), beyond which the effect on acoustic comfort would saturate (Experiment 1) or drop (Experiments 2 and 3). It can be concluded that, whereas some absorption is desirable, too much absorption—causing higher costs—might yield no further improvement, or may even decrease it (presumably because at some point the inner yard would lose its room acoustic quality; i.e., its “roominess”).

**Figure 12:**
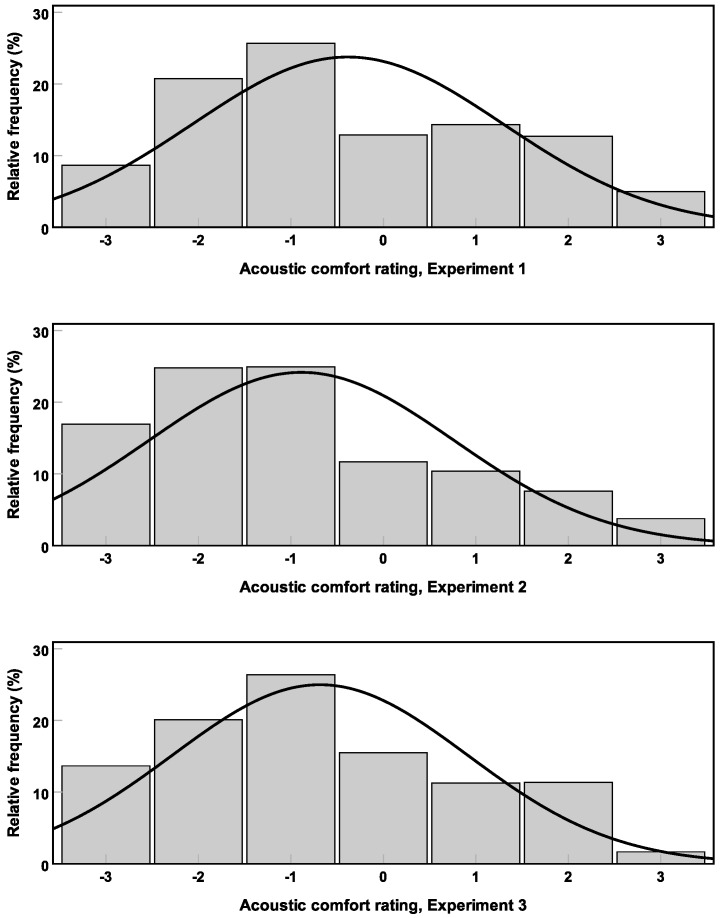
Acoustic comfort rating histograms for the data from Experiments 1 (**above**), 2 (**middle**) and 3 (**below**). Relative frequency is shown in percent.

With respect to the sound sources, all three experiments led to a consistent distinction between relatively unpleasant (e.g., **basketball**) and relatively pleasant (e.g., **children**) sound sources. Whereas in each experiment the unpleasant and the pleasant sounds were associated with lower and higher acoustic comfort ratings, the exact rating range was not robust between the three experiments. That might have to do with the SPL. The mean $L_{\mathrm{Aeq}}$ for Experiments 1, 2, and 3 was 52, 60, and 56 dB(A), respectively. Acoustic comfort rating histograms for the three experiments are shown in Figure 12. Although the median rating in all three experiments was −1, the mean rating was −0.4, −0.9, and −0.7 for Experiments 1, 2, and 3, respectively. That is, with increasing mean $L_{\mathrm{Aeq}}$ of the experiments, mean acoustic comfort decreased.

This is consistent with the fact that, for almost all sources and experiments, a tendency was observed, whereby increased $L_{\mathrm{Aeq}}$ (caused by reflection or by the distance to the observer) was associated with lower comfort ratings. Only for Experiment 1 (with the lowest mean $L_{\mathrm{Aeq}}$), and only in the case of the two rather pleasant **children** sounds, acoustic comfort increased with increasing $L_{\mathrm{Aeq}}$, which is similar to observations by Yang and Kang [15] for pleasant sounds. The results indicate that pleasant sounds can be used to increase perceived comfort in inner yards. It should be noted, however, that no definitive pleasant or unpleasant sounds exist. Pleasantness or unpleasantness is a subjective perceived quality of a sound, which can only be interpreted in the context under investigation [5]. Therefore, the pleasantness or unpleasantness of the sound in this study might be judged differently in other contexts and by other subjects.

In addition to the playing **children** sounds used in this study, Kang and Zhang [16] reported that natural and culture-related sounds (e.g., music) were preferred compared to artificial sounds. Furthermore, Hong and Jeon [42] reported that, in particular, water and bird sounds have been usually evaluated as the most effective and favorable sounds to improve urban sound environments [37,51].

The above discussion (on $L_{\mathrm{Aeq}}$) suggests three points for such experiments:

- Generally, increasing $L_{\mathrm{Aeq}}$ would be associated with decreased acoustic comfort.
- In order to use the bipolar scale appropriately, it is important that an appropriate (moderate) $L_{\mathrm{Aeq}}$ range is chosen. Too many stimuli with high $L_{\mathrm{Aeq}}$ (or equivalently a high mean $L_{\mathrm{Aeq}}$) can cause positive skewness and would not allow subjects to use the scale properly.
- If a high mean $L_{\mathrm{Aeq}}$ is not avoidable, it might be more appropriate to switch to a unipolar (negative) scale, such as the 11-point ICBEN annoyance scale [49]. This might be more practical, as subjects would more likely experience discomfort (rather than comfort).
- Although using a lower $L_{\mathrm{Aeq}}$ range (e.g., mean $L_{\mathrm{Aeq}}$ = 45 dB(A)) might be tempting and would probably lead to higher acoustic comfort, the ecological validity of the experiment should be taken into account. Note that the stimuli of this study were not calibrated, but normalized to the A-weighted level of the signal with the largest maximum absolute amplitude and this marks one of the limitations of this study.

With respect to observer position, Experiments 1 and 2 did not reveal a clear picture. Whereas balcony 2 was rated as the least comfortable observer position in Experiment 1, it was rated as more comfortable than balcony 0 in Experiment 2. Nevertheless, a few conclusions can be made based on the results. First, there are differences in acoustic comfort in distinct observer positions. This is in accordance with observations by Calleri et al. [27], in which different SPLs and different perceived wideness were reported in different observation positions and facades (including interactions similar to those in Experiment 1). Second, one factor for this is the SPL at the point of observation (see the discussion above). Third, post hoc listening to the stimuli revealed that stimuli with too much echo and flutter echo were rated significantly lower (i.e., more negatively) than those with low or moderate echo. That is, observer positions which were subject to echo and flutter echo were least comfortable.

Covering balcony ceilings with absorbers was not found to significantly affect acoustic comfort. However, a non-significant tendency was found, whereby, in the absence of absorbers on the facade, absorbing ceilings were more comfortable than reflecting ceilings. One explanation for this could be that, in comparison to the large facade surface, balcony ceilings are small. Hence, when absorbers were installed on the facade, possible benefits from additional absorption of balcony ceilings were marginal. In the presence of reflecting facades, however, a low-cost absorption on the balcony might not only be helpful to reduce noise level [29,30,35,52], but also to improve acoustic comfort. This should be subject to further investigations.

While interpreting the results and making conclusions, it is important to consider limitations of this study. While controlled laboratory experiments are suitable for comparisons (such as for this study), on-site studies offer a more natural, realistic and complex picture of the existing soundscape. Furthermore, as the subjects were seated in the laboratory, no visual modelling of the buildings and the inner yards was offered to them. It should be noted that visualizations and aural-visual interactions can affect sound and noise perception as well as perceived acoustic comfort [17,53,54,55,56,57]. Whereas with the chosen setup such visual effects were not included in the design, at the same time, this enabled an investigation of the perceptual quality of acoustical characteristics without any visual confounders. A further limitation of this study is that, for each stimulus, a single source was present. The experimental design can (and should) be extended in further works with mixtures of background (e.g., vegetation and birds) sounds and foreground inner yard sounds and/or with mixtures of several (foreground) sound sources. Lastly, it should be noted that—other than changes in balconies’ absorption coefficient in Experiment 3—balconies’ geometry and design were not changed systematically in this study. Interesting information about influences of balconies on acoustics can be found in [29,30,31,32,33,34,35].

In the analysis of the experimental results, a discussion of room acoustical parameters (beyond the SPL; i.e., early decay time, reverberation time, speech transmission index, clarity index, and etc.) has been avoided here. However, it is possible (and planned) to carry out a post hoc analysis of the associations between the room acoustical parameters and acoustic comfort, e.g., by means of correlations between each room acoustical parameter and the observed acoustic comfort. It is expected that room acoustical parameters can describe or predict the acoustic comfort to some degree [26,27,58,59].

## 8. Conclusions

Three laboratory experiments were reported which investigated short-term acoustic comfort and discomfort in housing complexes when relatively pleasant and unpleasant everyday life sound sources were present in virtual inner yards. The results showed that moderate absorption of the building facade increased acoustic comfort. Different sound sources and distinct observer positions were associated with different acoustic comfort. While relatively pleasant sounds were associated with comfort, discomfort was observed in the presence of relatively unpleasant sounds. Observer positions with strong echo and/or flutter echo and those at which high sound pressure levels were registered were found to be least comfortable. The results indicate that a careful acoustic design of the facades of housing complexes might be helpful to improve acoustic comfort of the residents. A further analysis of the results is needed and planned with regard to room acoustical parameters to identify which room acoustical parameters are good indicators of acoustic comfort.

## Abbreviations

The following abbreviations are used in this manuscript:

AIC	Akaike information criterion
ANOVA	Analysis of variance
BIC	Bayesian information criterion
H0	Null hypothesis
ICBEN	International Commission on Biological Effects of Noise
$L_{\mathrm{Aeq}}$	A-weighted equivalent continuous sound pressure level
$L_{\mathrm{AFmax}}$	A-weighted FAST response maximum sound pressure level
LSD	Fisher’s protected least significant difference
SPL	Sound pressure level
